# Early vaccination protects against childhood leukemia: A systematic review and meta-analysis

**DOI:** 10.1038/s41598-017-16067-0

**Published:** 2017-11-22

**Authors:** Mostafa Ebraheem Morra, Nguyen Dang Kien, Ahmed Elmaraezy, Omar Ayman M. Abdelaziz, Ahmed Lotfy Elsayed, Oday Halhouli, Ahmed Mosaad Montasr, Tran Le-Huy Vu, Chau Ho, Amr Sayed Foly, Anh Phan Phi, Wessam Magdy Abdullah, Marina Mikhail, Elizabeth Milne, Kenji Hirayama, Nguyen Tien Huy

**Affiliations:** 1 Faculty of Medicine, Al Azhar University, Cairo, 11884 Egypt; 2 Department of Obstetrics and Gynecology, Thai Binh University of Medicine and Pharmacy, Thai Binh, 30000 Vietnam; 3 Faculty of Medicine, Cairo University, Cairo, 11562 Egypt; 4 Faculty of Medicine, Zagazig University, Zagazig, 44519 Egypt; 5 University of Jordan, Faculty of Medicine, Amman, 11942 Jordan; 6 Faculty of Medicine, Tanta University, Tanta, 31527 Egypt; 7 University of California, Los Angeles, CA 90095 United States; 8 Hoan My Cuu Long Hospital, Can Tho, 900000 Vietnam; 9 Faculty of Medicine, Cairo University, Cairo, 11562 Egypt; 10 Department of Pharmacology, Loyola University Chicago, Illinois, 60546 USA; 11 Faculty of Medicine, Cairo University, Cairo, 11562 Egypt; 12 Department of Dermatology, Cairo University Hospital, Cairo, 11562 Egypt; 13 Telethon Kids Institute, The University of Western Australia, Crawley, Australia; 14 Department of Immunogenetics, Institute of Tropical Medicine (NEKKEN), Leading Graduate School Program, and Graduate School of Biomedical Sciences, Nagasaki University, 1-12-4 Sakamoto, Nagasaki, 852-8523 Japan; 15 Evidence Based Medicine Research Group & Faculty of Applied Sciences, Ton Duc Thang University, Ho Chi Minh City, Vietnam; 16 Department of Clinical Product Development, Institute of Tropical Medicine (NEKKEN), Leading Graduate School Program, and Graduate School of Biomedical Sciences, Nagasaki University, Nagasaki, Japan

## Abstract

Leukemia is the most commonly diagnosed childhood cancer, although its etiology is still largely unknown. Growing evidence supports a role for infection in the etiology of acute lymphocytic leukemia (ALL), and the involvement of the immune system suggests that vaccination may also play a role. However, the findings presented in the published literature are inconsistent. Therefore, we conducted a PRISMA systematic review and meta-analysis. 14 studies were identified and meta-analyzed. Vaccinations studied comprised Bacillus Calmette-Guérin (BCG) vaccine, Triple vaccine, Hepatitis B vaccine (HBV), Polio, Measles, Rubella, Mumps, trivalent MMR vaccine and *Haemophilus influenza* type B (HiB) vaccine. We observed a protective association between any vaccination in the first year of life and risk of childhood leukemia (summary odds ratio (OR) 0.58 [95% confidence interval (CI) 0.36–0.91]). When individual vaccines were analysed, some evidence of an association was seen only for BCG (summary OR 0.73 [95% CI 0.50–1.08]). In conclusion, early vaccination appears to be associated with a reduced risk of childhood leukemia. This finding may be underpinned by the association observed for BCG. Given the relatively imprecise nature of the results of this meta-analysis, our findings should be interpreted cautiously and replicated in future studies.

## Introduction

Leukemia is the most commonly diagnosed cancer in childhood, representing 31% of all cancer diagnoses^1^. Acute lymphocytic leukemia (ALL) and acute myelogenous leukemia (AML) are the most common subtypes. ALL alone accounts for 26% of cancers diagnosed from birth until four years of age. On the other hand, chronic leukemia is considered to be very rare in this age group^2^. Despite the great success in the treatment of ALL where the survival of children has steadily improved reaching 85% in both the United States and Europe, its etiology is still largely unknown^3^. A recent study by Ward *et al*. showed that the incidence of childhood acute leukemia in the United States had an annual increase rate of 0.7 percent for ALL and 1.1 percent for AML between 1975 and 2010^2^. However, the cause of this rising incidence has not been identified. Improved diagnostic techniques and improved notifications may partly explain these observed increases.

Various risk factors for childhood leukemia have been identified, such as ionizing radiation during prenatal and postnatal life^4,5^, paternal smoking^6–8^, exposure to household pesticides^9^ and benzene^10^. On the other hand, early exposure to infections may be protective for childhood ALL. This was originally postulated by Greaves in 1997 when he stated that both the pattern and timing of infections in early life is critical to the developmental programming of the immune system^11^. These immunomodulatory changes start with the passage of the mother’s immunoglobulins through the placenta in early pregnancy and in breast milk which is considered to be the first exposure to bacteria and viruses. He argued that current lifestyles in developed countries characterized by less exposure to infections or early weaning prevent mothers from sufficiently providing immune modulation to their offspring. Moreover, the improvement of hygiene in developed countries decreased the exposure of children themselves to infections. Therefore, when the inappropriately programmed immune system in a genetically predisposed child is exposed to common microbes in an abnormal time frame, it causes an abnormal immunological response thus increasing the risk of leukemia^11^. A recent meta-analysis of 14 case-control studies using day-care attendance as an indicator of the increased likelihood of early exposure to infections has shown a strong protective association between day care attendance and risk of ALL^12^. The growing evidence in support of the role of infection in the etiology of childhood leukemia has led to an interest in a possible role of childhood vaccination.

Published studies have reported contradictory results; studies from USA^13–18^, Canada^19,20^ and Finland^21,22^ have reported a reduced risk of leukemia with Bacille Calmette–Guerin (BCG) vaccination. However, a study in New Zealand^23^ showed no effect. After a thorough review of the literature, we aimed to systematically review and meta-analyze the current literature to provide evidence regarding the association between a range of vaccines and the risk of childhood leukemia.

## Material and Methods

### Search Strategy

Our study was conducted according to the recommendations of the PRISMA statement^24^ (Supplementary Table 1). We registered the protocol at PROSPERO with the registration number CRD42015024816. In June 14^th^, 2015, an electronic search was conducted in nine medical databases/search engines: PubMed, Scopus, Google Scholar, ISI Web of science, WHO Global health library, POPLINE, VHL, NYAM (New York academy of medicine) and SIGLE (System for information on grey literature in Europe). The descriptor used for searching in eight databases, except Google Scholar was: “(immunization or vaccines or vaccine or vaccination or vaccinated or immunized) and (risk or risks or association or correlation or odds) and (leukemia or leukaemia or leukemic or leukaemic) and (child or children or childhood or baby or infant or infants or newborn or neonates or pediatrics or pediatric)”. In Google Scholar, we used the keywords as the exact phrases “childhood leukemia”/ “childhood leukaemia” and “immunization, vaccines, vaccine, vaccination, vaccinated, immunized” with the option with at least one of the words and selected “where my words occur in the title of the article”. For every database, all variants spelled with “s” instead of “z” were covered in the search strategy. In addition, we manually searched the reference lists of relevant articles.

### Selection criteria

Studies that reported the association of early vaccination with leukemia in children under 20 years old and vaccines were included in this review with no restriction to language. Exclusion criteria were: (i) unreliably extracted data; (ii) leukemia cases reported in adults; (iii) overlapped data sets; (iv) abstract-only articles, review articles, theses, conference proceedings, books, and case reports. All studies identified were reviewed independently for eligibility by three authors and conflicts were resolved by seeking a discussion and consensus with senior researchers (NTH, EM, KH).

### Data extraction

Three independent reviewers extracted the data from the included studies. Any discrepancies were resolved by discussion to reach a consensus or through consulting senior researchers. A form for data extraction was designed for the authors to use. Sample: (author, title, year of publication, journal name, name of the study, study design, country, duration of follow up, sample size, prevalence, age, gender, type of leukemia (eg. ALL, AML, lymphoblastic, myeloblastic), exposure assessment, statistical analysis and risk estimation through the reported odds ratios (OR) and relative risks (RR). Where these had not been estimated and only raw numbers of participants were provided in tables, we estimated crude ORs from the information available.

### Quality assessment

The risk of bias of clinical trial^25,26^ was independently assessed by two reviewers using “The Cochrane Collaboration’s tool”. It consisted of six specific domains, including: sequence generation, allocation concealment, blinding of participants and personnel, incomplete outcome data, selective outcome reporting, and other sources of bias. The assessment of each domain was categorized as ‘low risk,’ ‘high risk,’ or ‘unclear risk’ of bias by two independent reviewers. Any differences between reviewers were resolved by discussion and by consultation from supervisor to reach a consensus if needed. Methodological quality assessment of observational studies^14,15,17,19,22,23,27–32^ was performed by using the risk of bias assessment tool of the “Cancer Council Australia”^33^. The metrics of this tool were as follows: subject selection (which included sub-metrics for case selection, control selection, and adequacy of case definition), comparability of groups on demographic characteristics and important potential confounders, ascertainment of exposure/treatment, and follow-up.

### Statistical methods

Quantitative synthesis with meta-analysis was performed using Comprehensive Meta-analysis software version 2.0 (Biostat, New Jersey, USA). Summary odds ratios (ORs) and 95% confidence intervals [CIs] were computed for individual vaccines when there were two or more studies providing results for the association between that specific vaccine and risk of childhood leukemia. Heterogeneity between studies was assessed using the Q statistic and I-squared tests. Heterogeneity was considered statistically significant if the P-value was < 0.1 or I-squared value was > 50%^34,35^. Random and fixed effects models were used depending on the heterogeneity among the studies.

When there were ten or more studies of a particular vaccine, we performed meta regression for moderators to examine the potential effects of study characteristics on the summary effect measure. Subgrouping of early/late and type of vaccination was performed to examine the effect of age at vaccination and type of infection on risk of leukemia. The effect of study characteristics on the summary OR was considered significant when the p-value was less than 0.05^36^. Publication bias was evaluated by funnel plot and Egger’s regression where there were five or more studies assessing the effect of a particular variable. Publication bias was considered significant when the P-value was < 0.1. The Duvall and Tweedie’s trim-and-fill method was used to increase symmetry by adjusting for studies that appeared to be missing in the presence of publication bias^37–39^.

## Results

### Literature search and study characteristics

**Figure 1:**
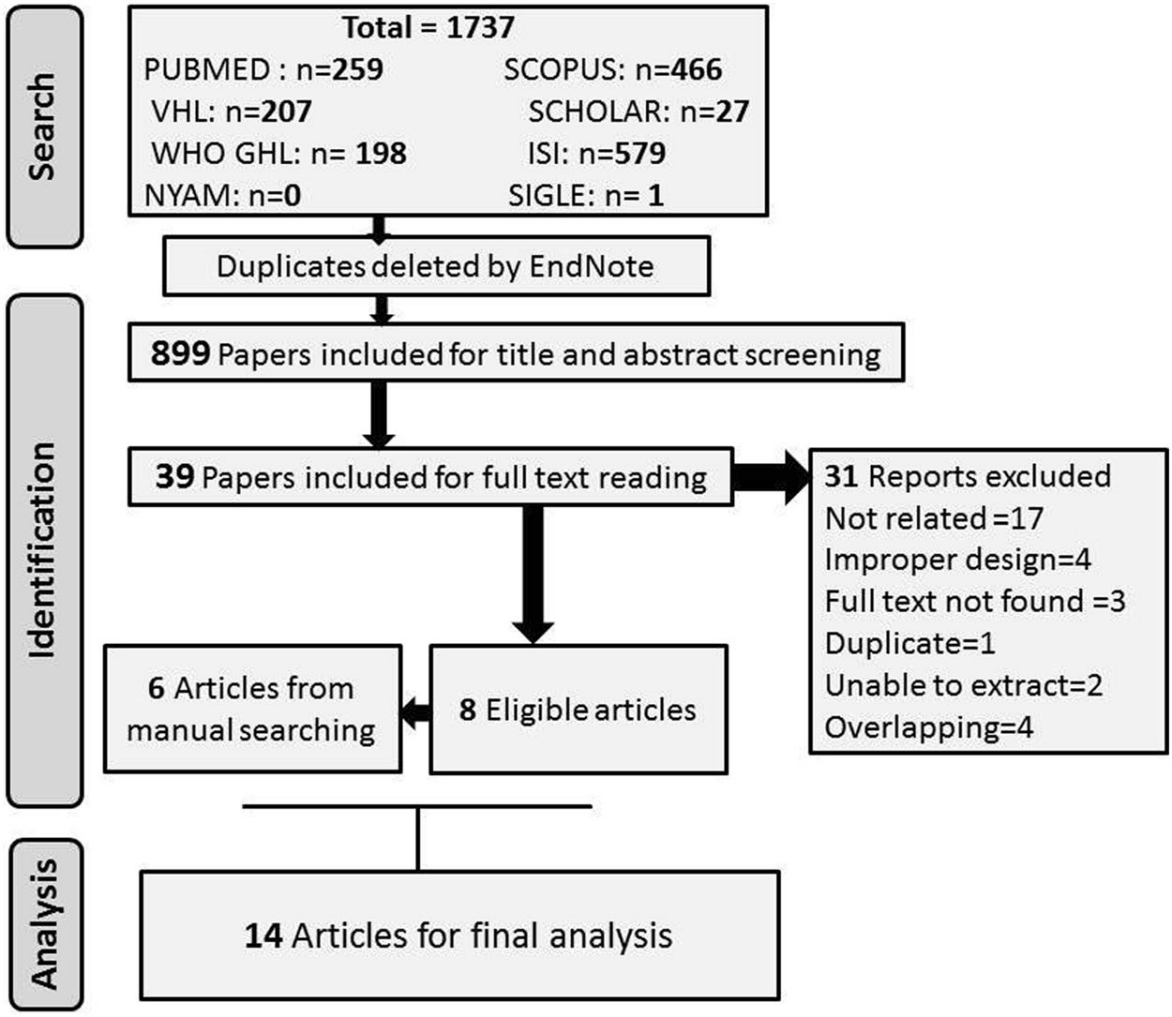
PRISMA flow diagram of studies’ screening and selection.

A total of 899 reports were included for title and abstract screening after automatic removal of duplicates in author, title, and date with Endnote software, and 39 articles were considered for full text reading. Only eight articles matched our selection criteria. The manual search of references also provided additional six reports. Among studies which were excluded from the manual search, there were two studies that did not provide a non-leukemic group^40,41^, two studies enrolled patients at more than 20 of age^42,43^, one study had overlapping participants^44^ and one study was a conference paper^45^. Hence, the total number of articles for final analysis was 14. Detailed characteristics of included studies are shown in Table 1. Twelve of the included studies were observational^14,15,17,19,22,23,27–32^, and two were clinical trials^25,26^ (Fig. 1). The results of meta-analysis of childhood leukemia and different types of vaccines are shown in Table 2.

**Table 1:** Basic characteristics of included studies.

Author/Year/Ref	Country	Leukemia			Vaccination	
		Type	Number	Method of Diagnosis	Type	Time
Crispen/1976	USA	ND	85356	ND	BCG	ND
Dockerty/1999	New Zealand	ALL	121	ND	BCG, MMR, Measles, Rubella, Oral Polio, DPT, Double vaccine, Hepatitis B	BCG: 3 months, 6 years, Measles: within 1 year
Nishi/1989	Japan	ALL	63	ND	BCG and Measles	1^st^ and 2^nd^ year
Groves/1999	USA	ALL	439	ND	MMR,Oral Polio, DPT, Tetanus, Diphteria, Hib polysaccharide and conjugate, Hepatitis B	ND
Von Kries/2000	Germany	AL	129	ND	BCG, MMR	BCG: at birth, MMR: after 1 year
MacArthur/2007	Canada	ND	399	Hematology, Pathology	Mumps, Measles, Rubella, Diphtheria, Pertussis,Tetanus, Polio, Hepatitis, BCG, and others	1^st^ and 2^nd^ year
Mallol.Mesnard/2007	France	AML, ALL, AL	726	Bone marrow analysis	BCG, Diphtheria, Tetanus, Poliomyelitis, Pertussis, Hepatitis B, Hib, Pneumococcus, Meningococcus, Measles, Mumps, Rubella	1^st^ and 2^nd^ year
Ma/2005	USA	ND	323	ND	DTP, MMR, Polio, Hepatitis B, and Hib	ND
Petridou/1997	Greece	ND	153	ND	DTP, BCG, Virus vaccines	At birth
Salonen/1976	Finland	ND	368	Pathology, Clinical	BCG	1–18 year
Comstock/1975	USA	ND	98	ND	BCG	ND
Davignon/1971	Canada	ND	96	ND	BCG	ND
Máthé/1974	France	ND	130	ND	BCG	ND
Sutherland/1982	England	ND	28	ND	BCG, vole-bacillus	ND

**Table 2:** Meta-analysis of the association of childhood leukemia with different types of vaccines from fourteen included studies.

Type of vaccines	No. of study	Total sample size (Leukemia/Control)	Heterogeneity		Model	Association with leukemia		Egger’s 2-tailed bias p-value
			p-value	*I* ^2^		p-value	Odds ratio (95% CI)	
Early vaccination	7	3000/3719581	0.0001	77.9	Random	0.019	0.58 (0.36–0.91)	0.60
BCG vaccines	12	3778/3760815	0.0001	77.7	Random	0.117	0.73 (0.50–1.08)	0.47
Mumps vaccines	4	3431/4999	0.98	0	Fixed	0.95	1.01 (0.82–1.23)	0.54
Hib vaccines	3	2528/4004	0.001	84.6	Random	0.83	1.06 (0.64–1.76)	0.89
HBV vaccines	5	2266/3906	0.67	0	Fixed	0.95	1.00 (0.85–1.18)	0.69
Triple vaccines	4	1986/2714	0.2	35.1	Fixed	0.56	1.10 (0.79–1.53)	0.99
Poliosip vaccines	4	2390/2769	0.99	0	Fixed	0.93	0.98 (0.70–1.39)	0.29
MMR vaccines	4	2302/2667	0.98	0	Fixed	0.78	0.96 (0.75–1.24)	0.85
Measles vaccines	6	3715/5627	0.02	59.1	Random	0.81	1.02 (0.85–1.23)	0.23
Rubella vaccines	5	3548/5267	0.96	0	Fixed	0.98	1.00 (0.82–1.23)	0.10

^I^Pooled odds ratios (OR) with corresponding 95% confidence intervals (95% CI) of the pooled results were calculated where more than one study had investigated the factor.

### Quality assessment

Using the Cochrane collaboration’s assessment tool, the two clinical trials^25,26^ were of unclear risk of bias regarding allocation concealment and sequence generation while the domain of incomplete outcome data were at low risk. According to Cancer Council Australia’s quality assessment tool of case control studies^14,15,17,19,22,23,27–32^, at least two out of six domains were at low risk of bias for all case control studies. Every article has more than three domains were at low risk of bias except for the study of Nishi *et al*.^30^. However, the overall quality of evidence in this systematic review is low and the results should be interpreted cautiously (Supplementary Table 2A and Supplementary Table 2B).

### Early vaccination

**Figure 2:**
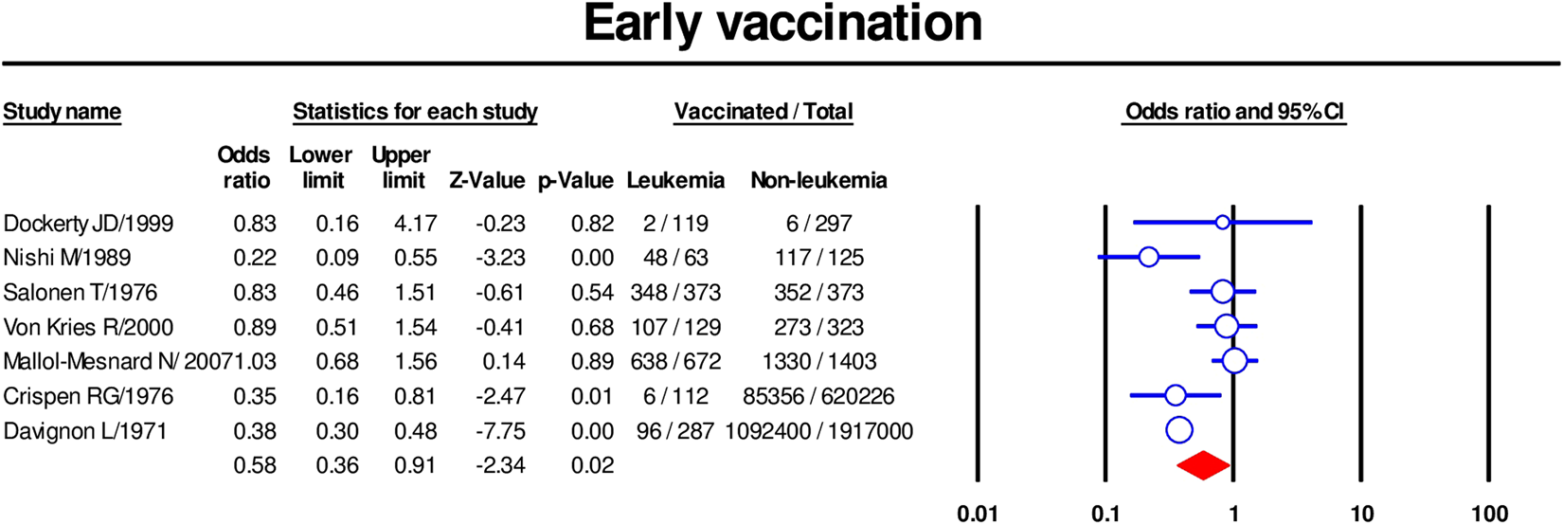
Forest plot showing results of meta-analysis of association of early vaccination with childhood leukemia. Cl: confidence interval.

The association between childhood leukemia and early vaccination, which included BCG vaccine, DPT vaccine, double vaccine and HBV vaccine given within the first year of life, was meta-analyzed. The summary ORs for receiving any type of vaccine in the first year of life (based on seven studies)^14,22,23,27,28,30,32^ was 0.58 [95% CI 0.36–0.91] (Fig. 2). There was significant heterogeneity among included studies (p < 0.1, I^2^ = 78%,), but no publication bias was seen (Supplementary Fig. 1); Egger’s test P value was 0.3.

### BCG vaccine

**Figure 3:**
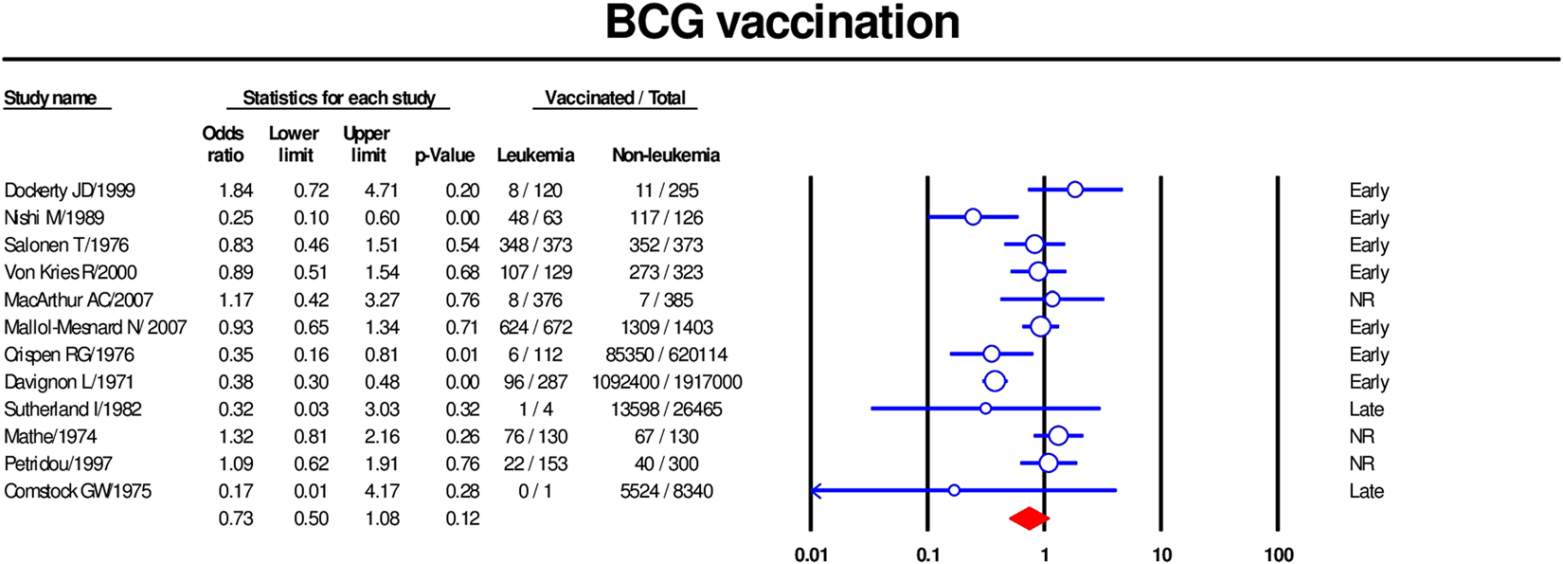
Forest plot showing results of meta-analysis of association of BCG vaccination with childhood leukemia. Cl: confidence interval.

**Figure 4:**
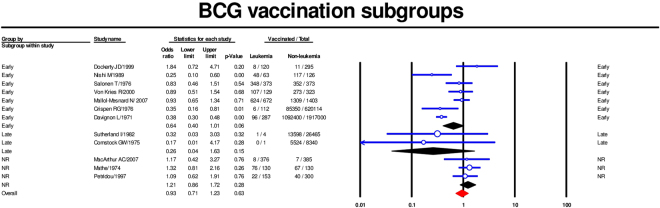
Forest plot showing subgroup analysis of association between early BCG vaccination and childhood leukemia. Cl: confidence interval.

Twelve studies were included in the meta-analysis of the association between BCG vaccine and childhood leukemia^14,19,22,23,25–32^. Using a random-effects model, the summary OR was 0.73 [95% CI 0.50–1.08] (Fig. 3). Our subgroups analysis by age at BCG vaccination revealed that both early vaccination (less than one year old) (summary OR 0,64; 95% CI [0.4–1.01])^14,22,23,27,28,30,32^ and later vaccination (summary OR 0.26; 95% CI 0.04–1.63)^25,26^ were associated with a lower risk of childhood leukemia, although the latter estimate lacked precision as there were only two studies. There was no evidence of association when the timing of vaccination was not reported^19,29,31^ (Fig. 4). A meta-regression revealed little effect of publication year on the association of BCG vaccination and childhood leukemia (coefficient 0.02, 95% CI [−0.005, 0.045], p value = 0.06) (Supplementary Fig. 2).

### Mumps vaccine

Meta-analysis of five studies showed no evidence of any association between mumps vaccination and childhood leukemia (summary OR 1.01 [95% CI 0.82–1.23])^15,17,19,23,28^. However, mumps vaccine was given alone in only one study^28^ and in a combined vaccination in four others^15,17,19,23^ so we could not perform a subgroup analysis (Supplementary Fig. 3).

### HiB vaccine

Meta-analysis of three studies^15,17,28^ revealed no association between HiB vaccination and childhood leukemia (summary OR 1.06 [95% CI 0.64–1.76]) (Supplementary Fig. 4).

### HBV vaccine

Four data sets were included in the meta-analysis of the association between HBV vaccination and childhood leukemia^17,19,23,28^. The result indicated that there was no association (summary OR 1.00 [95% CI 0.85–1.16]) (Supplementary Fig. 5).

### Triple vaccine

Triple vaccine is a combination of diphtheria, tetanus and pertussis (DPT) vaccines. Meta-analysis of four studies^15,17,23,31^ revealed no association between Triple vaccine and childhood leukemia risk (summary OR 1.1 [95% CI 0.79–1.53]) (Supplementary Fig. 6).

### Polio sip vaccine

Four datasets were included in the meta-analysis of the association between Polio vaccination and childhood leukemia risk^19,24,28,29^. The summary OR was 0.98 [95% CI 0.70–1.39] (Supplementary Fig. 7).

### MMR vaccine

Four studies were included in the meta-analysis of the association between trivalent MMR vaccine and childhood leukemia^19,24,28,29^. No association was seen (summary OR 0.96 [95% CI 0.75–1.24]) (Supplementary Fig. 8).

### Measles vaccine

There were six studies of the association between measles vaccination and childhood leukemia^15,17,19,23,28,30^. They provided seven datasets, as one study^23^ reported measles vaccine given both alone and in trivalent vaccine of MMR. No significant association between measles vaccine and leukemia was found (summary OR 0.96 [95% CI 0.71–1.30]). A subgroup meta-analysis was performed to investigate whether the association was affected by type of injection (alone or combined). No evidence of an association was seen in either subgroup (summary ORs were 0.84 [95% CI 0.39–1.83] and 1.02 [95% CI 0.66–1.59] respectively) (Supplementary Fig. 9).

### Rubella vaccine

Meta-analysis of six datasets available from five studies^15,17,19,23,28^ revealed no evidence of an association between rubella vaccine and leukemia risk (summary OR 1.00 [95% CI 0.82–1.23]). Subgroup analysis indicated that rubella vaccination was not associated with leukemia risk whether given alone or in combined trivalent MMR vaccine (Supplementary Fig. 10).

## Discussion

Our meta-analysis indicated that receiving any type of vaccine in the first year of life may be protective against childhood leukemia. When specific types of vaccine were separately analyzed, however, only the analysis of BCG revealed at least some evidence of a reduced risk. Because BCG is given early, it was not possible in this study to separate any potential protective effect of early vaccination from BCG vaccination itself. However, a protective association with BCG cannot be ruled out, given that no other individual vaccine was associated with a reduced risk of leukemia in this meta-analysis.

The protective association we observed between early vaccination (and/or BCG) and risk of childhood leukemia overall may involve similar biological pathways to those underlying the apparent reduction in leukemia risk associated with day-care attendance^12^. Similar to the potential for common infections during day-care attendance, exposure of children to microbial pathogen in vaccines early in life may mimic the role of infections in triggering the response of the naïve immune system. Schmiegelow *et at* proposed the hypothalamus-pituitary-adrenal axis hypothesis in which infections at early stage of life may alter plasma cortisol levels^46^. This can result in elimination of leukemic cells and reduce the risk of ALL^46,47^. Moreover, there is evidence that vaccines, both viral and bacterial, also have the ability to raise the plasma cortisol^48–51^. Thus, the adrenal hypothesis may explain in immunological terms the association between early vaccination and risk of leukemia. Such a mechanism would also be consistent with Greaves’ delayed-infection hypothesis^52^. However, this does not explain why BCG – but none of the other specific (mostly viral) vaccines – appeared to be associated (albeit weakly) with a reduction in leukemia risk.

Heterologous (or “off-target”) effects of BCG vaccination in children have been consistently observed^53,54^; outcomes for which there is some evidence of a protective include all-cause infant mortality^32,53^ and childhood cancer^32^. The biological pathways involved are unclear, but are thought to involve lymphocyte activation and/or innate immune memory that could promote protection beyond the intended target pathogen. Potential mechanisms are discussed in detail by Goodridge *et al*.^53^. In addition, many animal studies have reported beneficial effects of BCG in enhancing the immune response to unrelated pathogens^55^ and tumours. Theorically and practically, BCG vaccine has been utilized as an immunotherapy for a certain cancer^56–58^. The mechanism in which BCG vaccine ameliorates effectively cancer was not well-explored. Several studies investigated the alteration of different types of cytokines by BCG vaccine^59–62^, such as Th1’s cytokines (IFN-γ, TNF-α, IL-12) and Th2’s cytokines (IL-10, IL-4). These substances play roles in regulation of natural killer cells, macrophages or cytotoxic T lymphocytes cells^61^, which have antitumor effects. In addition, the antitumor activity of BCG vaccine through activating human dendritic cells, an antigen-present cells, has been studied^63–67^. This process was mediated by modulation of several cytokines such as IL2, IL10 and IFN gamma^67^. Taking specific antileukemia effect into consideration, studies were performed and their findings were promising. Jang *et al*.^65^ investigated the activity of dendritic cells of pediatric leukemia patients exposed by BCG, the result revealed an enhanced proliferation and maturation status. Recently, a BCG-derived heat shock protein, named HSP70, was investigated. Findings from animal and *in vitro* studies demonstrated an elevated immunogenicity effects against leukemia cells^68,69^.

### Strengths

To the best of our knowledge, this is the first comprehensive systematic review and meta-analysis investigating the association between vaccination and childhood leukemia. All steps of this systematic review and meta-analysis were conducted in strict accordance with the Cochrane handbook of systematic reviews^70^ and reported according to the recommendations of the PRISMA statement^24^. Moreover, stratification according to time of vaccination (for BCG vaccination) and type of vaccination (with early vaccination, Measles vaccine and Rubella vaccines) was further examined.

### Limitations

Although our systematic literature search was conducted on nine electronic libraries, the number of studies included in the final analysis was limited and it was not possible to conduct our meta-analysis by leukemia subtype. Furthermore, most of the included studies were observational. We were unable to evaluate the effects of several confounders such as prenatal and postnatal care, the nutrient supplies, and the microorganism exposure on the pooled results due to the lack of such information in the included studies. Further, some of the observational studies provided raw data only, from which we estimated crude study-specific ORs. Observational studies are subject to biases such as selection and recall bias. Except for Salonen 1976^22^ which was at moderate risk of bias, the six remaining studies were at high risk of bias. However, these biases (if present) would most likely have similarly affected the results for all types of vaccinations, not just early vaccination and/or BCG itself. We acknowledge that the association seen with BCG vaccination was not statistically significant; however, this was likely due to a lack of statistical power and a relevant protective association remains a possibility. While seven studies reported early vaccination, four studies were before 1990 and only two were in 21^st^ century; this led to some heterogeneity. Most studies reporting BCG vaccines were conducted before the 1980s. The design of those studies was not appropriate for evaluating the effect of BCG vaccination in risk of leukemia, because clinical trials were predominantly designed for protecting against tuberculosis. Additionally, three of them, which contained a large number of participants, reported leukemia deaths rather than leukemia cases^14,26,27^.

The change in vaccine schedule is worthy of note. According to Center for Disease Control and Prevention (CDC), many vaccines have currently been used for children at birth, such as HBV, or within the first year, such as DTA, Rotavirus, or *Haemophilus influenza* type b. These changes may alter the potential effect of early vaccine on childhood leukemia risk. Further studies should investigate the impact of these shifts in vaccine policy.

## Conclusion

Our comprehensive systematic review and meta-analysis indicated that the early vaccination may reduce the risk of childhood leukemia, and this finding may be attributable to BCG vaccination. Replication of this finding is important, and its impact in the context of changing vaccination policy should be carefully evaluated.

## Electronic supplementary material

Supplementary Information

## References

[1] Society, A. C. *Childhood Leukemia*, http://www.cancer.org/acs/groups/cid/documents/webcontent/003095-pdf.pdf (2015).

[2] Ward E, DeSantis C, Robbins A, Kohler B, Jemal A (2014). Childhood and adolescent cancer statistics, 2014. CA: a cancer journal for clinicians.

[3] Greaves M (2006). Infection, immune responses and the aetiology of childhood leukaemia. Nature Reviews Cancer.

[4] Ron E, Preston DL, Mabuchi K, Thompson DE, Soda M (1994). Radiation research.

[5] Preston DL (2004). Effect of recent changes in atomic bomb survivor dosimetry on cancer mortality risk estimates. Radiation research.

[6] Chang JS (2009). Parental smoking and childhood leukemia. Methods Mol Biol.

[7] Milne E (2012). Parental prenatal smoking and risk of childhood acute lymphoblastic leukemia. Am J Epidemiol.

[8] Farioli A (2014). Tobacco smoke and risk of childhood acute lymphoblastic leukemia: findings from the SETIL case-control study. Cancer causes & control: CCC.

[9] Ma X (2002). Critical windows of exposure to household pesticides and risk of childhood leukemia. Environmental Health Perspectives.

[10] Cogliano VJ, Baan R, Straif K (2011). Updating IARC’s carcinogenicity assessment of benzene. Am J Ind Med.

[11] Greaves M (1997). Aetiology of acute leukaemia. The Lancet.

[12] Urayama KY, Buffler PA, Gallagher ER, Ayoob JM, Ma X (2010). A meta-analysis of the association between day-care attendance and childhood acute lymphoblastic leukaemia. International journal of epidemiology.

[13] Comstock G, Livesay V, Webster R (1971). Leukaemia and BCG: A controlled trial. The Lancet.

[14] Crispen R, Rosenthal S (1976). BCG vaccination and cancer mortality. Cancer Immunology, Immunotherapy.

[15] Groves FD (1999). Infant vaccinations and risk of childhood acute lymphoblastic leukaemia in the USA. Br J Cancer.

[16] Buckley JD (1994). Epidemiological characteristics of childhood acute lymphocytic leukemia. Analysis by immunophenotype. The Childrens Cancer Group. Leukemia.

[17] Ma X (2005). Vaccination history and risk of childhood leukaemia. International Journal of Epidemiology.

[18] Rosenthal R (1972). BCG vaccination and leukemia mortality. JAMA.

[19] MacArthur AC (2008). Risk of childhood leukemia associated with vaccination, infection, and medication use in childhood: the Cross-Canada Childhood Leukemia Study. Am J Epidemiol.

[20] Davignon L, Robillard P, Lemonde P, Frappier A (1970). BCG vaccination and leukaemia mortality. The Lancet.

[21] Salonen T, Saxén L (1975). Risk indicators in childhood malignancies. International journal of cancer.

[22] Salonen T (1976). Prenatal and perinatal factors in childhood cancer. Ann Clin Res.

[23] Dockerty JD (1999). Infections, vaccinations, and the risk of childhood leukaemia. Br J Cancer.

[24] Moher D, Liberati A, Tetzlaff J, Altman DG (2009). Preferred reporting items for systematic reviews and meta-analyses: the PRISMA statement. Annals of internal medicine.

[25] Comstock GW, Martinez I, Livesay VT (1975). Efficacy of BCG vaccination in prevention of cancer. Journal of the National Cancer Institute.

[26] Sutherland I (1982). BCG and vole bacillus vaccination in adolescence and mortality from leukaemia. Statistics in medicine.

[27] Davignon L, Lemonde P, St-Pierre J, Frappier ABCG (1971). vaccination and leukaemia mortality. Lancet (London, England).

[28] Mallol-Mesnard N (2007). Vaccination and the risk of childhood acute leukaemia: the ESCALE study (SFCE). International journal of epidemiology.

[29] Mathe G, Facy F, Hatton F, Halle-Pannenko O (1974). BCG vaccination and acute leukaemia. Biomedicine / [publiee pour l’A.A.I.C.I.G.].

[30] Nishi M, Miyake H (1989). A case-control study of non-T cell acute lymphoblastic leukaemia of children in Hokkaido, Japan. J Epidemiol Community Health.

[31] Petridou E (1997). The risk profile of childhood leukaemia in Greece: a nationwide case-control study. Br J Cancer.

[32] Von Kries R, Peter Grunert V, Kaletsch U, Michaelis J, Göbel U (2000). Prevention of childhood leukemia by BCG vaccination in newborns? A population-based case-control study in Lower Saxony, Germany. Pediatric hematology and oncology.

[33] Olver, I. *et al*. *Development of Clinical Practice Guidelines Using Cancer Council Australia’s Cancer Guidelines Wiki*. (2014).

[34] Zintzaras E, Lau J (2008). Synthesis of genetic association studies for pertinent gene–disease associations requires appropriate methodological and statistical approaches. Journal of clinical epidemiology.

[35] Higgins JP, Thompson SG, Deeks JJ, Altman DG (2003). Measuring inconsistency in meta-analyses. BMJ: British Medical Journal.

[36] Munafo MR, Flint J (2004). Meta-analysis of genetic association studies. TRENDS in Genetics.

[37] Begg, C. B. & Mazumdar, M. Operating characteristics of a rank correlation test for publication bias. *Biometrics*, 1088–1101 (1994). 7786990

[38] Peters JL, Sutton AJ, Jones DR, Abrams KR, Rushton L (2006). Comparison of two methods to detect publication bias in meta-analysis. Jama.

[39] Egger M, Smith GD, Schneider M, Minder C (1997). Bias in meta-analysis detected by a simple, graphical test. Bmj.

[40] Härö A (1985). The effect of BCG-vaccination and tuberculosis on the risk of leukaemia. Developments in biological standardization.

[41] Ambrosch F, Wiedermann G, Krepler P (1986). Studies on the influence of BCG vaccination on infantile leukemia. Developments in biological standardization.

[42] Kendrick MA, Comstock GW (1981). BCG vaccination and the subsequent development of cancer in humans. Journal of the National Cancer Institute.

[43] Villumsen M (2009). Risk of lymphoma and leukaemia after bacille Calmette-Guerin and smallpox vaccination: a Danish case-cohort study. Vaccine.

[44] Snider DE, Comstock GW, Martinez I, Caras GJ (1978). Efficacy of BCG Vaccination in Prevention of Cancer: An Update: Brief Communication2. JNCI: Journal of the National Cancer Institute.

[45] Rosenthal SR (1986). Cancer precursors and their control by BCG. Developments in biological standardization.

[46] Schmiegelow K, Vestergaard T, Nielsen SM, Hjalgrim H (2008). Etiology of common childhood acute lymphoblastic leukemia: the adrenal hypothesis. Leukemia.

[47] Azevedo-Silva F, Camargo B, Pombo-de-Oliveira MS (2010). Implications of infectious diseases and the adrenal hypothesis for the etiology of childhood acute lymphoblastic leukemia. Brazilian journal of medical and biological research = Revista brasileira de pesquisas medicas e biologicas.

[48] El Yousfi M (2005). The inflammatory response to vaccination is altered in the elderly. Mechanisms of ageing and development.

[49] Oken E, Kasper DL, Gleason RE, Adler GK (1998). Tetanus toxoid stimulation of the hypothalamic-pituitary-adrenal axis correlates inversely with the increase in tetanus toxoid antibody titers. The Journal of clinical endocrinology and metabolism.

[50] Pourcyrous M, Korones SB, Crouse D, Bada HS (1998). Interleukin-6, C-reactive protein, and abnormal cardiorespiratory responses to immunization in premature infants. Pediatrics.

[51] Rowe J (2005). Th2-Associated Local Reactions to the Acellular Diphtheria-Tetanus-Pertussis Vaccine in 4- to 6-Year-Old Children. Infection and Immunity.

[52] Greaves M (1988). Speculations on the cause of childhood acute lymphoblastic leukemia. Leukemia.

[53] Goodridge HS (2016). Harnessing the beneficial heterologous effects of vaccination. Nature reviews. Immunology.

[54] Pollard AJ, Finn A, Curtis N (2017). Non-specific effects of vaccines: plausible and potentially important, but implications uncertain. Archives of Disease in Childhood.

[55] Freyne B, Marchant A, Curtis N (2015). BCG-associated heterologous immunity, a historical perspective: experimental models and immunological mechanisms. Transactions of the Royal Society of Tropical Medicine and Hygiene.

[56] Boehm BE (2017). Efficacy of bacillus Calmette-Guerin Strains for Treatment of Nonmuscle Invasive Bladder Cancer: A Systematic Review and Network Meta-Analysis. The Journal of urology.

[57] Kamat AM (2017). Society for Immunotherapy of Cancer consensus statement on immunotherapy for the treatment of bladder carcinoma. Journal for immunotherapy of cancer.

[58] Redelman-Sidi G, Glickman MS, Bochner BH (2014). The mechanism of action of BCG therapy for bladder cancer–a current perspective. Nature reviews. Urology.

[59] Janaszek-Seydlitz W (2014). Effect of different Bacillus Calmette-Guerin substrains on growth inhibition of T24 bladder cancer cells and cytokines secretion by BCG activated peripheral blood mononuclear cells of PBMCs. Advances in clinical and experimental medicine: official organ Wroclaw Medical University.

[60] Kleinnijenhuis J (2014). Long-lasting effects of BCG vaccination on both heterologous Th1/Th17 responses and innate trained immunity. Journal of innate immunity.

[61] Liu X (2014). Cytokines as effectors and predictors of responses in the treatment of bladder cancer by bacillus Calmette-Guerin. Future oncology (London, England).

[62] Portevin D, Young D (2013). Natural killer cell cytokine response to M. bovis BCG Is associated with inhibited proliferation, increased apoptosis and ultimate depletion of NKp44(+)CD56(bright) cells. PloS one.

[63] Sanarico N (2011). *Different transcriptional profiles of* human monocyte-derived dendritic cells infected with distinct strains of Mycobacterium tuberculosis and Mycobacterium bovis bacillus Calmette-Guerin. Clinical & developmental immunology.

[64] Thurnher M (1997). Bacillus Calmette-Guerin mycobacteria stimulate human blood dendritic cells. International journal of cancer.

[65] Yang J (2010). Effect of Bacillus Calmette-Guerin on the expansion of dendritic cells from peripheral blood of pediatric patients with leukemia *in vitro*. Zhongguo shi yan xue ye xue za zhi.

[66] Kim KD (1999). Enhanced antigen-presenting activity and tumour necrosis factor-alpha-independent activation of dendritic cells following treatment with Mycobacterium bovis bacillus Calmette-Guerin. Immunology.

[67] Cheadle EJ, Selby PJ, Jackson AM (2003). Mycobacterium bovis bacillus Calmette-Guerin-infected dendritic cells potently activate autologous T cells via a B7 and interleukin-12-dependent mechanism. Immunology.

[68] Jimbo J (2008). Induction of leukemia-specific antibodies by immunotherapy with leukemia-cell-derived heat shock protein 70. Cancer science.

[69] Ye Q, Wang ZH, Qin SK (2008). Preparation of heat shock protein 70 (Hsp70) and idiotypic determinant single-chain antibody (Id-ScFv) in a patient with B-cell chronic lymphatic leukemia (B-CLL) and antitumor effect of peptide complex Hsp70-Id. Ai zheng = Aizheng = Chinese journal of cancer.

[70] Collaboration, T. C. *Cochrane Handbook for Systematic Review of Intervention*s. (2011).

