# Supplementary material for: Early vaccination protects against childhood leukemia: A systematic review and meta-analysis
Source: Sci Rep. 2017 Nov 22;7:15986. doi: 10.1038/s41598-017-16067-0 (PMC5700199; doi:10.1038/s41598-017-16067-0)
Supplement: Supplementary file 1 — Supplementary Information [file 41598_2017_16067_MOESM1_ESM.pdf]

Early vaccination protects against childhood leukemia: A systematic review and meta-analysis

Mostafa Ebraheem Morra[1,⊕], Nguyen Dang Kien[2,⊕], Ahmed Elmaraezy[1, ⊕], Omar Ayman M Abdelaziz[3], Ahmed Lotfy Elsayed[4], Oday Halhouli[5], Ahmed Mosaad Montasr[6], Tran Le-Huy Vu[7], Chau Ho[8], Amr Sayed Foly[9], Anh Phan Phi[10], WessamMagdy Abdullah[11], Marina Mikhail[12], Elizabeth Milne[13, *], Kenji Hirayama[14], Nguyen Tien Huy[15,16*]

Supplementary Table 1: PRISMA 2009 checklist of items to include when reporting a systematic review or meta-analysis[1].

Section/topic	#	Checklist item	Reported on page #
**TITLE**			
Title	1	Identify the report as a systematic review, meta-analysis, or both.	Page 1
**ABSTRACT**			
Structured summary	2	Provide a structured summary including, as applicable: background; objectives; data sources; study eligibility criteria, participants, and interventions; study appraisal and synthesis methods; results; limitations; conclusions and implications of key findings; systematic review registration number.	Page 4
**INTRODUCTION**			
Rationale	3	Describe the rationale for the review in the context of what is already known.	Page 5
Objectives	4	Provide an explicit statement of questions being addressed with reference to participants, interventions, comparisons, outcomes, and study design (PICOS).	Page 6
**METHODS**			
Protocol and registration	5	Indicate if a review protocol exists, if and where it can be accessed (e.g., Web address), and, if available, provide registration information including registration number.	Page 6
Eligibility criteria	6	Specify study characteristics (e.g., PICOS, length of follow-up) and report characteristics (e.g., years considered, language, publication status) used as criteria for eligibility, giving rationale.	Page 7
Information sources	7	Describe all information sources (e.g., databases with dates of coverage, contact with study authors to identify additional studies) in the search and date last searched.	Page 6
Search	8	Present full electronic search strategy for at least one database, including any limits used, such that it could be repeated.	Page 6
Study selection	9	State the process for selecting studies (i.e., screening, eligibility, included in systematic review, and, if applicable, included in the meta-analysis).	Page 6-7

Data collection process	10	Describe method of data extraction from reports (e.g., piloted forms, independently, in duplicate) and any processes for obtaining and confirming data from investigators.	Page 7-8
Data items	11	List and define all variables for which data were sought (e.g., PICOS, funding sources) and any assumptions and simplifications made.	Page 8
Risk of bias in individual studies	12	Describe methods used for assessing risk of bias of individual studies (including specification of whether this was done at the study or outcome level), and how this information is to be used in any data synthesis.	Page 9-10
Summary measures	13	State the principal summary measures (e.g., risk ratio, difference in means).	NA
Synthesis of results	14	Describe the methods of handling data and combining results of studies, if done, including measures of consistency (e.g., I^2) for each meta-analysis.	Page 8

**Supplementary Table 2A: Quality assessment of clinical trials studies**						
**Studies**	**Domain of Assessment**					
	**Sequence Generation**	**Allocation Concealment**	**Blinding**	**Incomplete Outcome Data**	**Selective Outcome Reporting**	**Other**
**Comstock/1975**	high	unclear	high	low	unclear	low
**Sutherland /1982**	low	unclear	low	low	unclear	low

Supplementary Table 2B: Quality assessment of case control studies

Studies	Domain of Assessment						
	Subject Selection						
**Author/year/ref**	**Cases**	**Adequacy of case definition**	**Controls**	**Comparability of groups**	**Ascertainment of exposure/treatment**	**Follow-up**	**Overall**
**Dockerty/1999**	2	2	2	2	0	1	High
**Crispen/1976**	2	1	2	2	0	1	High
**Nishi/1989**	1	2	2	0	0	0	High
**Groves/1999**	2	2	2	2	0	0	High
**Von Kries/2000**	2	2	2	2	2	0	high
**MacArthur/2008**	2	2	2	2	0	1	High
**Mallol-Mesnard /2007**	2	2	2	2	0	1	High
**Ma/2005**	2	2	2	2	0	1	Moderate
**Petridou/1997**	1	2	2	2	2	1	High
**Salonen /1976**	2	2	2	2	0	1	Moderate
**Davignon/1971**	2	2	2	2	0	0	High
**Máthé /1974**	2	1	2	2	0	0	High

Supplementary Figure 1: Funnel plot of publication bias assessment in the meta-analysis of association between early vaccination and childhood leukemia.

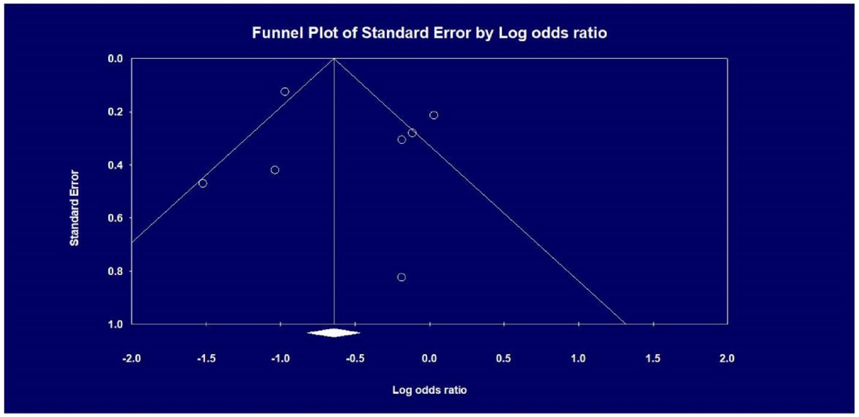

Supplementary Figure 2: The scatter-plot showing meta-regression of childhood leukemia on year of publication of BCG vaccination studies.

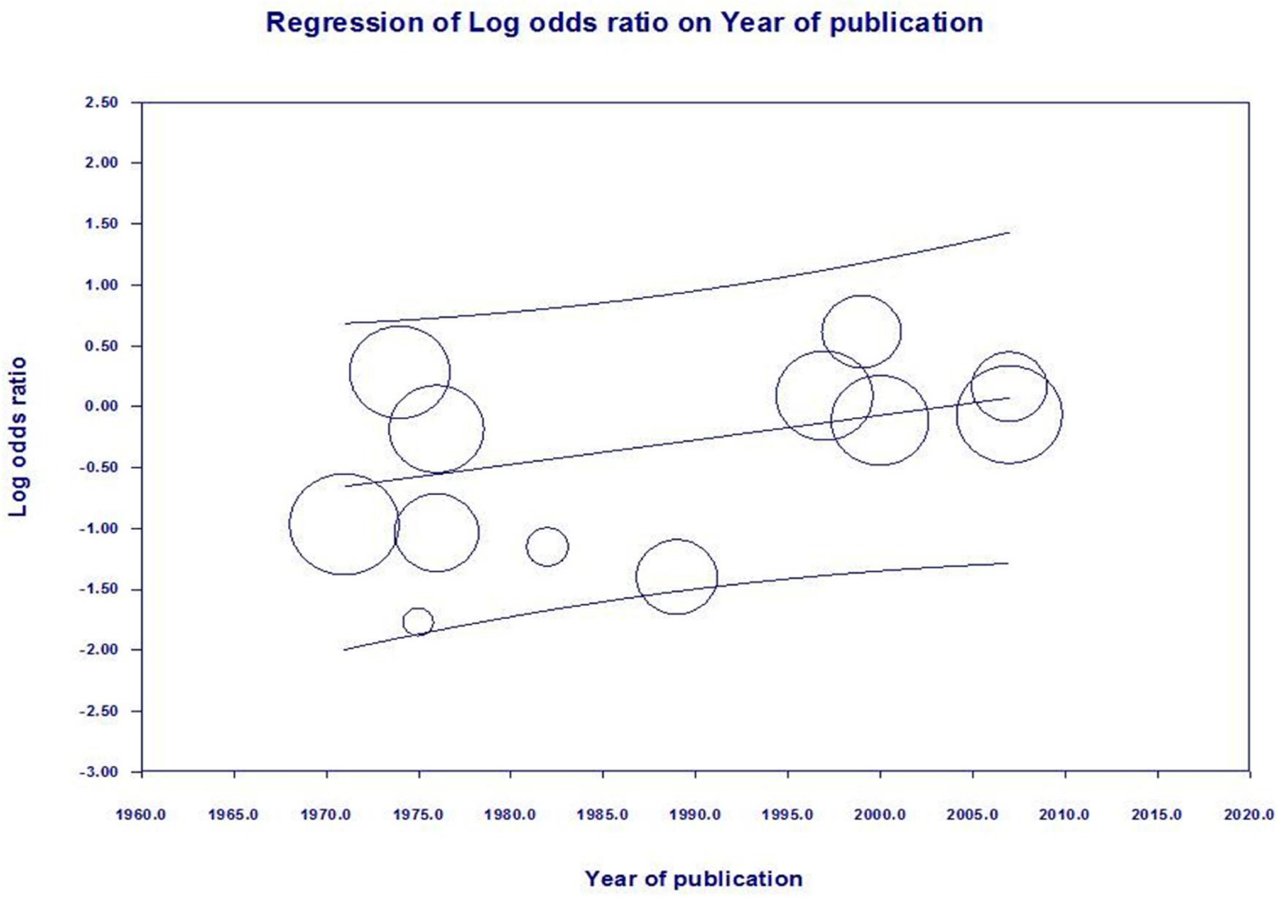

Supplementary Figure 3: Forest plot showing meta-analysis of mumps vaccination and subgroups meta-analysis.

Mumps vaccination

Study name	Odds ratio	Lower limit	Upper limit	Z-Value	p-Value	Vaccinated / Total: Leukemia	Vaccinated / Total: Non-leukemia
Dockerty JD/1999	1.02	0.39	2.70	0.04	0.97	6 / 112	15 / 285
Groves FD/1999	1.03	0.66	1.59	0.11	0.91	395 / 439	394 / 439
Ma X/2005	0.92	0.52	1.61	-0.31	0.76	299 / 323	381 / 409
MacArthur AC/2007	0.93	0.62	1.40	-0.35	0.73	334 / 388	346 / 398
Mallol-Mesnard N/ 2007	1.08	0.78	1.50	0.46	0.65	539 / 596	1103 / 1229
	1.01	0.82	1.23	0.06	0.95		

Odds ratio and 95% CI

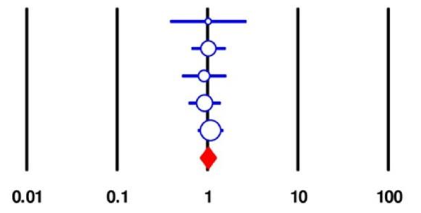

Supplementary Figure 4: Forest plot showing meta-analysis of HiB vaccination and subgroups meta-analysis.

HiB vaccination

Study name	Odds ratio	Lower limit	Upper limit	Z-Value	p-Value	Vaccinated / Total: Leukemia	Vaccinated / Total: Non-leukemia
Groves FD/1999	0.79	0.61	1.03	-1.75	0.08	206 / 439	232 / 439
Ma X/2005	0.93	0.48	1.80	-0.23	0.82	306 / 323	389 / 409
Mallol Mesnard/2007	1.55	1.20	2.00	3.31	0.00	582 / 672	1132 / 1403
	1.06	0.64	1.76	0.22	0.83		

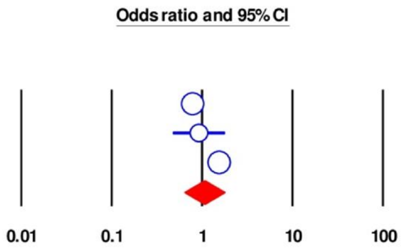

Supplementary Figure 5: Forest plot showing meta-analysis of rubella vaccination and subgroups meta-analysis.

HBV vaccination

Study name	Statistics for each study					Vaccinated / Total	
	Odds ratio	Lower limit	Upper limit	Z-Value	p-Value	Leukemia	Non-leukemia
Dockerty JD/1999	1.03	0.67	1.60	0.15	0.88	62 / 114	151 / 282
MacArthur AC/2007	1.17	0.42	3.27	0.31	0.76	8 / 376	7 / 385
Mallol-Mesnard N/ 2007	1.04	0.86	1.26	0.38	0.71	250 / 672	510 / 1403
Ma X/2005	0.75	0.45	1.23	-1.14	0.25	289 / 323	376 / 409
	1.00	0.85	1.18	0.06	0.95		

Odds ratio and 95% CI

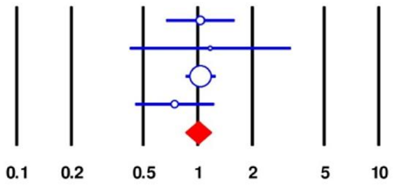

Supplementary Figure 6: Forest plot showing meta-analysis of triple vaccination and subgroups meta-analysis.

Triple vaccination

Study name	Odds ratio	Lower limit	Upper limit	Z-Value	p-Value	Leukemia (Vaccinated / Total)	Non-leukemia (Vaccinated / Total)
Dockerty JD/1999	0.94	0.59	1.49	-0.26	0.79	80 / 118	197 / 285
Groves FD/1999	0.73	0.33	1.60	-0.79	0.43	424 / 439	428 / 439
MA X/2005	1.05	0.23	4.74	0.07	0.95	320 / 323	405 / 409
Petridou E/1997	1.94	1.03	3.64	2.06	0.04	139 / 153	251 / 300
	1.10	0.79	1.53	0.57	0.57		

Odds ratio and 95% CI

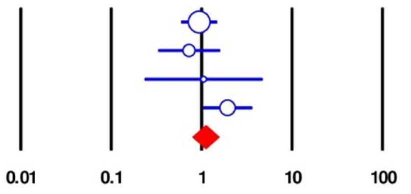

Supplementary Figure 7: Forest plot showing meta-analysis of polio sip vaccination and subgroups meta-analysis.

Polio sip vaccination

Study name	Statistics for each study					Vaccinated / Total	
	Odds ratio	Lower limit	Upper limit	Z-Value	p-Value	Leukemia	Non-leukemia
Dockerty JD/1999	0.95	0.60	1.52	-0.20	0.85	80 / 117	197 / 284
Groves FD/1999	1.10	0.46	2.62	0.22	0.83	429 / 439	428 / 439
Ma X/2005	1.05	0.23	4.74	0.07	0.95	320 / 323	405 / 409
MacArthur AC/2007	0.97	0.47	2.00	-0.09	0.93	371 / 386	384 / 399
	0.98	0.70	1.39	-0.09	0.93		

Odds ratio and 95% CI

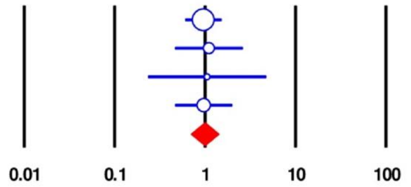

Supplementary Figure 8: Forest plot showing meta-analysis of MMR vaccination and subgroups meta-analysis.

MMR vaccination

Study name	Statistics for each study					Vaccinated / Total	
	Odds ratio	Lower limit	Upper limit	Z-Value	p-Value	Leukemia	Non-leukemia
Dockerty JD/1999	1.02	0.39	2.70	0.04	0.97	6 / 112	15 / 285
Groves FD/1999	1.03	0.66	1.59	0.11	0.91	395 / 439	394 / 439
Ma X/2005	0.92	0.52	1.61	-0.31	0.76	299 / 323	381 / 409
MacArthur AC/2007	0.93	0.62	1.40	-0.35	0.73	334 / 388	346 / 398
	0.96	0.75	1.24	-0.28	0.78		

Odds ratio and 95% CI

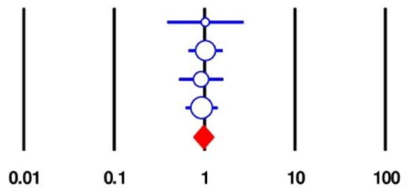

Supplementary Figure 9: Forest plot showing meta-analysis of measles vaccination and subgroups meta-analysis.

Measles vaccination

Study name	Subgroup within study	Odds ratio	Lower limit	Upper limit	Z-Value	p-Value
		Statistics for each study				
Dockerty JD/1999 A	Alone	1.65	1.06	2.57	2.22	0.03
Dockerty JD/1999 C	MMR	1.02	0.39	2.70	0.04	0.97
MacArthur AC/2007	MMR	0.93	0.62	1.40	-0.35	0.73
Mallol-Mesnard N/ 2007	Alone	1.05	0.75	1.48	0.31	0.76
Ma X/2005	MMR	0.92	0.52	1.61	-0.31	0.76
Nishi M/1989	Alone	0.25	0.10	0.60	-3.08	0.00
Groves FD/1999	MMR	1.03	0.66	1.59	0.11	0.91
		1.02	0.85	1.23	0.25	0.80

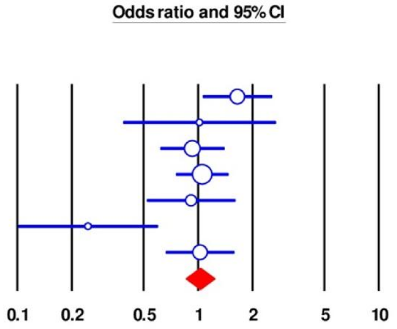

Measles vaccination subgroups

Group by Subgroup within study	Study name	Subgroup within study	Odds ratio	Lower limit	Upper limit	Z-Value	p-Value	Leukemia	Non-leukemia
			Statistics for each study					Vaccinated / Total	
Alone	Dockerty JD/1999 A	Alone	1.65	1.06	2.57	2.22	0.03	58 / 113	106 / 272
Alone	Mallol-Mesnard N/ 2007	Alone	1.05	0.75	1.48	0.31	0.76	541 / 596	1110 / 1229
Alone	Nishi M/1989	Alone	0.25	0.10	0.60	-3.08	0.00	48 / 63	117 / 126
Alone			1.09	0.84	1.40	0.64	0.52		
MMR	Dockerty JD/1999 C	MMR	1.02	0.39	2.70	0.04	0.97	6 / 112	15 / 285
MMR	MacArthur AC/2007	MMR	0.93	0.62	1.40	-0.35	0.73	334 / 388	346 / 398
MMR	Ma X/2005	MMR	0.92	0.52	1.61	-0.31	0.76	299 / 323	381 / 409
MMR	Groves FD/1999	MMR	1.03	0.66	1.59	0.11	0.91	395 / 439	394 / 439
MMR			0.96	0.75	1.24	-0.28	0.78		
Overall			1.02	0.85	1.23	0.25	0.80		

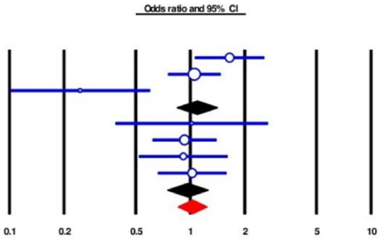

Supplementary Figure 10: Forest plot showing meta-analysis of rubella vaccination and subgroups meta-analysis.

Rubella vaccination

Study name	Subgroup within study	Odds ratio	Lower limit	Upper limit	Z-Value	p-Value	Vaccinated / Total: Leukemia	Vaccinated / Total: Non-leukemia
Dockerty JD/1999 A	Alone	0.45	0.05	3.90	-0.72	0.47	1 / 115	5 / 262
Dockerty JD/1999 C	MMR	1.02	0.39	2.70	0.04	0.97	6 / 112	15 / 285
Groves FD/1999	MMR	1.03	0.66	1.59	0.11	0.91	395 / 439	394 / 439
Ma X/2005	MMR	0.92	0.52	1.61	-0.31	0.76	299 / 323	381 / 409
MacArthur AC/2007	MMR	0.93	0.62	1.40	-0.35	0.73	334 / 388	346 / 398
Mallol-Mesnard N/ 2007	Alone	1.09	0.78	1.52	0.52	0.60	540 / 596	1104 / 1229
		1.00	0.82	1.23	0.03	0.98		

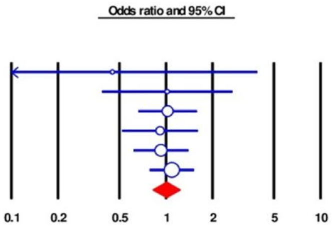

Rubella vaccination subgroups

Group by Subgroup within study	Study name	Subgroup within study	Odds ratio	Lower limit	Upper limit	Z-Value	p-Value
Alone	Dockerty JD/1999 A	Alone	0.45	0.05	3.90	-0.72	0.47
Alone	Mallol-Mesnard N/ 2007	Alone	1.09	0.78	1.52	0.52	0.60
Alone			1.07	0.77	1.48	0.40	0.69
MMR	Dockerty JD/1999 C	MMR	1.02	0.39	2.70	0.04	0.97
MMR	Groves FD/1999	MMR	1.03	0.66	1.59	0.11	0.91
MMR	Ma X/2005	MMR	0.92	0.52	1.61	-0.31	0.76
MMR	MacArthur AC/2007	MMR	0.93	0.62	1.40	-0.35	0.73
MMR			0.96	0.75	1.24	-0.28	0.78

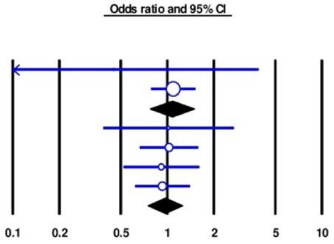

1 Moher, D., Liberati, A., Tetzlaff, J. & Altman, D. G. Preferred reporting items for systematic reviews and meta-analyses: the PRISMA statement. *Annals of internal medicine* **151**, 264-269 (2009).
